# The impact of migration on women’s mental health in the postpartum period

**DOI:** 10.1590/S1518-8787.2016050005617

**Published:** 2016-06-17

**Authors:** Lígia Moreira Almeida, Cristina Costa-Santos, José Peixoto Caldas, Sónia Dias, Diogo Ayres-de-Campos

**Affiliations:** I Instituto de Saúde Pública. Faculdade de Medicina da Universidade do Porto. Porto, Portugal; II Centro de Investigação em Tecnologias e Serviços de Saúde. Faculdade de Medicina da Universidade do Porto. Porto, Portugal; III Universidade de Fortaleza. Fortaleza, CE, Brasil; IV Instituto de Higiene e Medicina Tropical, Universidade Nova de Lisboa. Lisboa, Portugal; V Departamento de Obstetrícia e Ginecologia. Centro Hospitalar de São João. Faculdade de Medicina. Universidade do Porto. Porto, Portugal

**Keywords:** Transients and Migrants, Pregnant Women, Depression, Postpartum, Stress, Psychological, Life Change Events, Mental Health, Cross-Sectional Studies

## Abstract

**OBJECTIVE:**

To assess the influence of I mmigration on the psychological health of women after childbirth.

**METHODS:**

In this cross-sectional study, immigrant and Portuguese-native women delivering in the four public hospitals of the metropolitan area of Porto, Portugal, were contacted by telephone between February and December 2012 during the first postpartum month to schedule a home visit and fill in a questionnaire. Most immigrant (76.1%) and Portuguese mothers (80.0%) agreed to participate and with the visits, thus a total of 89 immigrants and 188 Portuguese women were included in the study. The questionnaire included the application of four validated scales: Mental Health Inventory-5, Edinburgh Postpartum Depression Scale, Perceived Stress Scale, and Scale of Satisfaction with Social Support. Statistical analysis included t-test and Chi-square or Fisher’s test, and logistic regression models.

**RESULTS:**

Immigrants had an increased risk of postpartum depression (OR = 6.444, 95%CI 1.858–22.344), and of low satisfaction with social support (OR = 6.118, 95%CI 1.991–18.798). We did not perceive any associations between migrant state, perceived stress, and impoverished mental health.

**CONCLUSIONS:**

Immigrant mothers have increased vulnerabilities in the postpartum period, resulting in an increased risk of postpartum depression and lesser satisfaction with the received social support.

## RESUMO

**OBJETIVO:**

Avaliar a influência da imigração na saúde psicológica da mulher após o parto.

**MÉTODOS:**

Neste estudo transversal, mulheres imigrantes e portuguesas com partos nos quatro hospitais públicos da região metropolitana de Porto, Portugal, foram contatadas por telefone entre fevereiro e dezembro de 2012, durante o primeiro mês pós-parto, para agendar uma visita domiciliar e preencher um questionário. A maioria das mães imigrantes (76,1%) e das mães portuguesas (80,0%) aceitou participar e aceder a visitas domiciliares, totalizando 89 imigrantes e 188 mulheres portuguesas incluídas no estudo. O questionário incluiu a aplicação de quatro escalas validadas: Inventário de Saúde Mental-5, Escala de Depressão Pós-parto de Edimburgo, Escala de *Stress* Percebido e Escala de Satisfação com o Suporte Social. As análises estatísticas incluíram os testes t-student, Qui-quadrado ou teste de Fisher e o cálculo de modelos de regressão logística.

**RESULTADOS:**

As imigrantes tiveram risco aumentado de depressão pós-parto (OR 6,444; IC95% 1,858–22,344) e de baixa satisfação com o suporte social (OR = 6,118; IC95% 1,991–18,798). Não houve associação entre migração, *stress* percebido e saúde mental empobrecida.

**CONCLUSÕES:**

Mães imigrantes apresentam vulnerabilidades aumentadas no período pós-parto, aumentando o risco de depressão pós-parto e havendo menor satisfação com o apoio social recebido.

## INTRODUCTION

The postpartum period is often difficult for the recent mother, since it requires large emotional and biophysical adjustments. Regardless of pregnancy as a normative period of a woman’s life, the postpartum phase carries increased health risks with it. Minor problems consequent to delivery, such as constipation and after pains resulting from uterine involution, may combine with more severe conditions to decrease maternal well-being^4^ ^,^ ^9^ ^,^ ^18^ ^,^ ^19^.

Several studies report that migrant women have a higher risk of complications during pregnancy and the postpartum period^14^. Being an immigrant or belonging to an ethnic minority is associated with a higher frequency of perinatal infection, increased perinatal and infant mortality, higher maternal mortality, greater number of preterm, and low birth-weight children^8^. Several studies indicate that about 20.0% of maternal deaths, directly or indirectly related to pregnancy, occur among women with scarce, delayed, or non-existent prenatal care^9^ ^,^ ^18^.

Migrant women also present a greater risk for mental illness, including depression, schizophrenia, and post-traumatic stress, because of the interaction of specific psychosocial determinants (e.g., forced migration and its psychological impact, generalized insecurity associated with refugees and asylum seekers, human trafficking, irregular status, and the tendency to integrate low income employment with scarce security conditions as well as some barriers to healthcare access – language, mobility, legal status, length of stay, country of origin, health care provider’s attitudes, and culture, besides occupational factors)^4^ ^,^ ^18^. These factors are likely to increase vulnerability during pregnancy and psychopathological complications before and after birth – postnatal depression and psychosis^4^ ^,^ ^6^. Migrant women frequently report sensations of insecurity, isolation, self-perception of affective deprivation from key relationships, longing for their own culture and family, strangeness to new cultural habits, linguistic challenges, religious differences, and sometimes even hostility and indifference from the local population^4^ ^,^ ^8^. Increased distress and anxiety frequently foster postpartum depression^9^ ^,^ ^13^ ^,^ ^19^.

The incidence of postpartum depression seems to be greater when the mother’s social network (e.g., family and friends) and social support are weak^10^ ^,^ ^13^. Women who are isolated, displaced, depressed, and without traditional references of support are more vulnerable to this condition^18^ ^,^ ^19^.

Several problems have been identified with the quality of health care provided during the postpartum period. We identified the delays in the initial contact when complications occur, and in the beginning of the treatment, shortness of financial resources to contact the population (migrants are often considered hard-to-reach population, and the public health systems not always have human resources available to contact this fringe of the population), and lack of evidence-based maternity care (because of real and perceived barriers, several migrant women still cannot access or receive a minimum health care attention in pregnancy) ^a^ ^,^ ^b^. Some studies report that public health facilities offer scarce support during this period, and women have difficulty in meeting their mental health care needs, even when health care is universally available^13^. A previous study conducted in our region (north of Portugal) showed that some immigrant and native women are unsatisfied with the medical attention received during the postpartum period^2^.

This study aimed at assessing the influence of immigration on the psychological health of women after childbirth. As the Portuguese national health system offers the same care to all women during pregnancy, irrespective of their documentation status, and has a very structured standardization of care, observed dissimilarities should theoretically be due to differences in the social support and quality of health care.

## METHODS

### Sampling and Recruitment

A cross-sectional observational study was carried out. Administrative databases of the four public maternity hospitals in the Porto metropolitan area (Hospital de Sao Joao, Centro Hospitalar de Vila Nova de Gaia e Espinho, Centro Hospitalar do Porto, and Hospital Pedro Hispano) were searched on a weekly basis between February and December 2012, to identify all births that occurred among immigrant mothers. The latter were defined as women born outside Portugal whose parents were also born outside Portugal, irrespective of their documentation status. To act as a comparison group, the two subsequent births registered in each of these hospitals to Portuguese native mothers were selected. The contact telephone numbers of all mothers were obtained from hospital records. Approval was obtained from the Ethics Committees of all participating hospitals.

### Instruments and Procedure

In the three to four weeks period following delivery, one of the researchers attempted to telephone all selected women. Participants were considered non-responders if they failed to answer three telephone calls (Immigrants = 18, Portuguese = 33). Regarding these refusals, a brief analysis on the narrow information that we could obtain allows to sustain, with reasonable certain, that those women present sociodemographic characteristics (e.g., maternal age, place of residence) similar to those who accepted to participate. Of those that answered, they were excluded from the study if they reported residing outside the Porto metropolitan area (Immigrants = 7, Portuguese = 3), if they reported a multiple birth (Immigrants = 3, Portuguese = 8), or if they indicated that they were giving their baby up for adoption (Immigrants = 0, Portuguese = 3). All remaining women were explained the aim of the study and were asked for informal consent to participate, and the researcher attempted to schedule a visit to their home or elsewhere of convenience, to answer a written questionnaire. From the total number of women selected from hospital records, 89 (76.1%) of immigrant mothers answered the phone, agreed to schedule a visit, and were visited, while this occurred in 188 (80.0%) of Portuguese mothers. A total of 277 answered questionnaires were obtained.

Home visits occurred in the subsequent weeks after scheduling – five to eight weeks after delivery (according to the commonly accepted definition of postpartum period)^3^. During the home visits, carried out by a single researcher, each participant received written and oral information on the study, and written consent to participate was obtained. Mothers were asked to fill in the questionnaire in the presence of the researcher, and whenever doubts about a question arose or a delay in response was noticed, the items were explained. Obstetrical data were complemented and confirmed with information from the mother’s pregnancy health book, a record of prenatal and intrapartum clinical care that is given to all pregnant women in Portugal.

We collected data on demographic characteristics, socioeconomic status, education level, income and employment status, household and family aggregate, lifestyles and health behaviors, gynecologic and obstetrical history, characterization of prenatal and intrapartum care (e.g., complications of pregnancy and labor), and postpartum medical attention (e.g., comorbidities, cultural health habits and practices – when applicable – and migration specific issues). Additionally, four specific validated scales were applied: Mental Health Inventory-5^16^, Edinburgh Postpartum Depression Scale^3^ ^,^ ^7^, Perceived Stress Scale^5^ ^,^ ^17^, and Scale of Satisfaction with Social Support^15^.

Free health care for all pregnant women, regardless of legal status, has been offered in Portugal since 2009. There are many local Primary Health Care Centers run by family physicians, and the system mandates first contact at this level, except in acute health conditions. For the latter, individuals have access to pre-hospital care and transport, or direct admission to emergency hospital services. Specialized care takes place in public hospitals on referral of the family physician. Primary Health Care Centers also develop local actions for the promotion of health, prevention of disease, vaccination, and rehabilitation, usually organized by nursing teams. Prenatal care in low-risk pregnancies is conducted in Primary Health Care Centers, while there are guidelines for the referral of pregnant women to specialized obstetric care. National guidelines also exist on the number of prenatal visits, laboratory evaluations, and ultrasound exams to be performed in low-risk pregnancy.

### Statistical Analysis

Collected data were organized and coded using IBM SPSS Statistics software, version 19.0 (Chicago, Illinois, United States). Regarding sociodemographic data, we used the t-Student and Chi-square tests, respectively, to analyze maternal age and to compare migrants and Portuguese-native women regarding maternal education, income, parity, and marital status.

Univariate analysis was performed (t-test and Chi-square or Fisher’s test) to compare the scores for mental health, perceived stress, social support, and postpartum depression in migrant and Portuguese women (data not shown). Conceptual and statistical criteria were used to construct subsequent multivariate models (logistic regression). The models considered all variables that met the criterion p < 0.2 in the univariate analysis, or if they were judged to be clinically or conceptually relevant to accomplish the aim of this study: to analyze the role of being a migrant (comparing immigrants and native women) in the frequency of perceived stress, depression, impoverished mental functioning, and perceived low social support at postpartum. The models were adjusted for variables frequently associated with pregnancy and postpartum complications: preterm birth and low birth weight, smoking habits before and during pregnancy, obstetric complications (e.g., gestational diabetes and hypertension disorders, congenital malformations, previous stillbirth and/or neonatal death, three or more spontaneous miscarriages), maternal age, and previous health conditions (e.g., anemia, depression, hypertension). For accomplishing the objective of this study, we also added the variable “being a migrant”.

In the logistic regression model, for mental health evaluation, due to lack of cases in each category, the variable “gestational age” was removed, and smoking in second trimester was replaced by smoking in pregnancy. For postpartum depression, to avoid collinearity, we used the variable “low birth weight”, removed the variable “preeclampsia”, and maintained the “gestational hypertension” and “depression prior to pregnancy” variables. We also added the variable “marital status”, because of the possible effect of living with partner on depression. For detection of stress, smoking in first trimester was replaced by “smoking in pregnancy” and, to avoid collinearity, we used the variable “low birth weight”, removed the variable “preeclampsia”, and maintained “gestational hypertension”. Finally, for social support we added the variables “marital status” and “birth weight”.

## RESULTS

In the immigrant group, 48 women (54.0%) originated from Brazil, 23 (26.0%) from eastern European countries, and 18 (20.0%) from Portuguese-speaking African countries. Mean length of stay in Portugal was 7.35 years (SD = 3.63). Legalization of the immigrant status had been obtained by 47 women (53.0%), while 36 (40.0%) stated that they were in the process of obtaining legal status, and six remained illegal.

Additional sociodemographic data are presented in Table 1. Immigrants showed maternal age significantly higher and were more likely to be multiparous and to have a monthly family income below 1,000€. No significant differences between the groups were found in marital status. Considering the years of school attendance, Portuguese women were equally distributed between seven to nine years, 12 years and higher education, while most migrants only completed 12 years of school.

**Table 1:** Sociodemographic data and parity of women in the postpartum period living at the metropolitan area of Porto, 2012.

Variables in the model	Migrants		Portuguese		Total		p
		
	(n = 89)		(n = 188)		(n = 277)		
		
	n	%	n	%	n	%	
Maternal age^a^	31	4.72	29	4.66	29	4.77	**0.001^c^**
Parity							**0.005^d^**
Primiparous	37	42.0	112	60.0	149	54.0	-
Multiparous	52	58.0	76	40.0	128	46.0	-
Marital status							0.720^d^
With partner	67	76.0	146	78.0	213	78.0	-
Without partner	21	24.0	41	22.0	63	23.0	-
Family income^b^ (€)							0.119^d^
< 500	26	29.0	34	18.0	60	22.0	-
500-1,000	39	44.0	75	40.0	114	42.0	-
1,001-1,500	12	14.0	43	23.0	55	20.0	-
1,501-2,000	9	10.0	25	13.0	34	12.0	-
> 2,000	3	3.0	10	5.0	13	5.0	-
Family income (€)							**0.018^d^**
≤ 1,000	65	73.0	109	58.0	174	63.0	-
> 1,000	24	27.0	78	42.0	102	37.0	-
Maternal education							**0.024^d^**
1-4 years	4	5.0	12	6.0	16	6.0	-
5-6 years	11	12.0	13	7.0	24	9.0	-
7-9 years	15	17.0	57	30.0	72	26.0	-
10-12 years	44	49.0	64	34.0	108	39.0	-
Higher education	15	17.0	42	22.0	57	21.0	-

^a^ n and standard deviation.

^b^ Regarding family income, when analyzing differences between classes, we considered that it would be useful to explore a new categorization of the variable, to counteract the possible lack of the predictive value of the sample when subdivided into five classes. Therefore, we also present the results of the new analysis.

^c^ t-student test.

^d^ Chi-square or Fisher’s exact test.

Significant values are presented in bold.

Table 2 displays the major influences on “perceived mental health”. The variables with significant odds for an impoverished postpartum adjustment were episiotomy and multiparity. Mothers with medium and higher education had a reduced risk, and immigrant status was not a statistically significant factor.

**Table 2:** Logistic regression model for Impoverished Maternal Mental Health (MHI-5, cut-off ≥ 13).

Variables in the model	OR^a^	95%CI
Migrant^b^	0.163	0.026–1.030
Maternal education		
1-4 years	-	-
5-6 years	0.708	0.052–9.550
7-9 years	0.132	0.010–1.772
10-12 years	0.021	**0.001–0.412**
Higher education	0.007	**0.000–0.665**
Family income^b^ (€)		
< 500	-	-
500-1,000	1.767	0.280–11.140
1,001-1,500	0.290	0.034–2.474
1,501-2,000	0.408	0.017–9.907
> 2,000	-	-
Parity (multiparous)	13.820	**1.895–100.789**
Marital status^b^ (living with partner)	0.214	0.040–1.148
Adverse obstetrical outcomes^b^ (previous pregnancies)	3.236	0.516–20.313
Depression^b^ (prior to pregnancy)	3.477	0.331–26.557
Non-gestational anaemia^b^	1.108	0.110–11.203
Smoking in pregnancy^c^		
Non-smoker	–	–
≤ 10	–	–
> 10	5.568	0.298–104.044
Delivery mode^b^		
Eutocic	–	–
Instrumented	0.543	0.055–5.400
Caesarean section	1.284	0.146–11.252
Metrorrhagia^b^	0.952	0.192–4.711
Placenta praevia^b^	6.563	0.299–143.858
Gestational hypertension^b^	3.490	0.501–24.294
Episiotomy (only vaginal delivery)	116.660	**10.021–1358.087**

^a^ odds ratio adjusted for all variables included (that met the inclusion criterion p > 0.2). Variables added: “being a migrant”; Variables removed: “gestational age”.

^b^ absent from predictive model.

^c^ mean of cigarettes/day.

Significant values are presented in bold.

Table 3 shows the major influences on “postpartum depression”. The variables with significant odds were migrant status, history of depression in prior pregnancy, gestational hypertension, adverse obstetric outcomes in previous pregnancies, and smoking more than 10 cigarettes per day during pregnancy. Cesarean section, family monthly income above 500€, and smoking less than 10 cigarettes per day during pregnancy appear to have a protective effect against depression.

**Table 3:** Logistic regression model for Postpartum Depression (EPDS, cut-off > 10).

Variables in the model	OR^a^	95%CI
Migrant	6.444	**1.858–22.344**
Maternal education^b^		
1-4 years	-	-
5-6 years	1.091	0.086–13.786
7-9 years	3.196	0.260–39.290
10-12 years	0.655	0.049–8.799
Higher education	2.501	0.137–45.585
Family income (€)		
< 500	-	-
500-1,000	0.200	**0.050–0.799**
1,001-1,500	0.163	**0.035–0.768**
1,501-2,000	0.011	**0.001–0.203**
> 2,000	-	-
Maternal age^b^	1.045	0.937–1.164
Parity^b^ (multiparous)	2.608	0.789–8.617
Marital status^b^ (living with partner)	0.749	0.243–2.309
Adverse obstetric outcomes (previous pregnancies)	4.086	**1.212–13.780**
Depression (before pregnancy)	101.859	**8.534–1215.710**
Non-gestational anaemia^b^	1.780	0.257–12.322
Gestational age		
Term	-	-
Preterm	4.227	0.746–23.967
Post-term	-	-
Infant’s low birth weight^b^	0.268	0.045–1.608
Smoking in pregnancy^c^		
Non-smoker	-	-
≤ 10	0.071	0.013–0.379
> 10	52.248	1.562–1747.627
Delivery mode		
Normal	-	-
Instrumented	1.839	0.430–7.871
Caesarean-section	0.054	**0.011–0.259**
Metrorrhagia^b^	0.287	0.067–1.237
Gestational hypertension	76.745	**13.255–444.347**
Gestational diabetes^b^	2.494	0.507–12.279

^a^ odds ratio adjusted for all variables included (that met the inclusion criterion p > 0.2). Variables removed: “preeclampsia” and “marital status”.

^b^ absent from predictive model.

^c^ mean of cigarettes/day.

Significant values are presented in bold.

Table 4 displays the major influences on “emotional stress”. The variables with significant odds were episiotomy, adverse obstetric outcomes in previous pregnancies, diagnosis of non-gestational anemia, low birth-weight, and gestational hypertension. Smoking less than 10 cigarettes a day during pregnancy, and attending school during 10-12 years appeared to have a protective effect against stress. Immigrant status was not a statistically significant factor.

**Table 4:** Logistic regression model for Perceived Stress Scale (PSS, cut-off > 26).

Variables in the model	OR^a^	95%CI
Migrant^b^	0.708	0.216–2.322
Maternal education		
1-4 years	-	-
5-6 years	0.408	0.035–4.732
7-9 years	0.232	0.022–2.415
10-12 years	0.062	**0.005–0.792**
Higher education	0.071	0.004–1.420
Family income^b^ (€)		
< 500	-	-
500-1,000	3.353	0.840–13.378
1,001-1,500	0.553	0.109–2.798
1,501-2,000	2.281	0.289–18.034
> 2,000	-	-
Parity^b^ (multiparous)	2.409	0.600–9.672
Marital Status^b^ (living with partner)	0.531	0.174–1.616
Adverse obstetric outcomes (previous pregnancies)	8.802	**1.911–40.530**
Depression^b^ (previous to pregnancy)	0.754	0.158–3.597
Anaemia (previous to pregnancy)	8.383	**1.633–43.024**
Infant with low birth weight	7.643	**1.953–29.919**
Smoking in pregnancy^c^		
Non-smoker	-	-
≤ 10	0.021	**0.001–0.293**
> 10	3.172	0.316–31.860
Delivery mode^b^		
Normal	-	-
Instrumented	0.671	0.118–3.817
Caesarean section	0.518	0.107–2.505
Infant with malformations^b^	5.653	0.614–52.036
Gestational hypertension	5.216	**1.160–23.443**
Gestational diabetes^b^	2.194	0.459–10.491
Episiotomy (only vaginal delivery)	18.820	**3.953–89.609**

^a^ odds ratio adjusted for all variables included (that met the inclusion criterion p > 0.2). Variables removed: “preeclampsia”.

^b^ absent from predictive model.

^c^ mean of cigarettes/day.

Significant values are presented in bold.

Table 5 provides the major influences on “satisfaction with social support”. The variables that had significant odds were migrant status, previous diagnosis of depression, postpartum hemorrhage, increased maternal age, episiotomy, multiparity, and hypertensive disorders of pregnancy. Urinary infections during pregnancy and family monthly income above 500€ had a protective effect.

**Table 5:** Logistic regression model for Perceived Lack of Social Support (SSSS, cut-off > 30).

Variables in the model	OR^a^	95%CI
Migrant	6.118	**1.991–18.798**
Maternal education^b^		
1-4 years	-	-
5-6 years	1.924	0.173–21.400
7-9 years	0.697	0.086–5.646
10-12 years	0.591	0.067–5.199
Higher education	1.654	0.136–20.161
Family income (€)		
< 500	-	-
500-1,000	0.221	**0.066–0.740**
1,001-1,500	0.060	**0.012–0.297**
1,501-2,000	0.118	**0.015–0.912**
> 2,000	-	-
Maternal age	1.147	**1.026–1.282**
Parity (multiparous)	3.766	**1.116–12.715**
Marital status^b^ (living with partner)	0.777	0.255–2.362
Adverse obstetric outcomes^b^ (previous pregnancies)	1.232	0.365–4.153
Depression (previous to pregnancy)	13.356	**2.318–76.963**
Anaemia^b^ (previous to pregnancy)	0.359	0.050– 2.564
Gestational age^b^		
Term	-	-
Preterm	0.642	0.051–8.144
Post-term	-	-
Infant birth weight^b^		
Normal	-	-
Low (< 2,500 g)	0.203	0.026–1.554
High (> 4,000 g)	1.567	0.223–11.030
Gestational hypertension	5.890	**1.186–29.239**
Gestational diabetes^b^	1.634	0.370–7.203
Metrorrhagia^b^	1.250	0.336–4.648
Urinary infection	0.143	**0.026–0.797**
Postpartum hemorrhage	8.936	**2.456–32.509**
Episiotomy (only vaginal delivery)	6.670	**2.322–19.158**

^a^ odds ratio adjusted for all variables included (that met the inclusion criterion p > 0.2). Variables added: “marital status” and “infant’s birth weight”.

^b^ absent from predictive model.

Significant values are presented in bold.

## DISCUSSION

Our results show that migrant status is associated with an increased odd of postpartum depression and of lower satisfaction with social support. On the other hand, it seems unrelated to perceived stress and mental health in the puerperium.

The design of this study has several strong points, such as the allowance of an accurate selection of immigrant women who participated, and a timely scheduling of home visits. The inclusion of all public hospitals in the area was decided to allow a good representation of the immigrant population, and the proportion of nationalities in the sample is very similar to that reported by the immigration authorities for the Porto area^10^.

Questionnaires were filled in at participants’ own pace and in surroundings that were comfortable to them, with the support of a trained researcher (Psychology graduate) who was uninvolved in the provision of health care. The presence of the researcher was also intended to help women with the clarification of concepts and in translation issues when answering the questionnaire.

One of the weaknesses of the study is the non-inclusion of births taking place in private hospitals and in home settings. Nevertheless, these account for only about 12.0% of all births in the area and are usually only chosen by the more affluent families, as neither are funded by the state.

Non-responders to telephone calls and those who declined a home visit are a possible source of bias in this study, as they may include women who cannot pay to keep their telephones active, women giving false telephone numbers at the hospital, those with a limited understanding of the Portuguese language, and those who may be uneasy in showing their living conditions. Cultural barriers and fear of being part of official statistics may also have driven undocumented immigrants away from the study. It is therefore likely that illegal immigrants are underrepresented in this sample. Regarding sampling, additional bias must be considered: 1:2 sampling approach does not ensure the representativeness of the exposure (migration) in population, but allowed to ensure a sufficient representation of migrants in the sample; 1:2 sampling was adopted to certify adequate statistical power as subsequent analyses or comparisons were considered important. Still, the limited sample size could have contributed to the lack of differences in the prevalence of preterm delivery, low-newborn weight, and fetal malformations reported in other studies^4^ ^,^ ^18^ ^,^ ^19^. Our concerns were gradually surpassed as logistic regression models progressively showed robustness of associations and odds calculations. The most important effect of the small sample size can be found in the confidence intervals associated with each odds ratio: its large amplitude is uninformative about the actual magnitude of odds variation.

External generalizability of the results must be considered with some caution, because the cut-offs applied are only validated for Portuguese population (with no information for migrants). This aspect is inevitably present in every study with this target population, irrespective of the country where is conducted, but is still an important bias to consider.

Regarding maternal mental health, explicitly the ability to maintain an adjusted mental functioning after delivery, we observed that several conditions and procedures contribute to deprive mothers’ emotional, psychological, and behavioral well-being, increasing anxiety, perceptions of losing control, and discouragement^4^ ^,^ ^9^. Some of these aspects respond to individual perceptions and subjective meanings attributed by women, assuming a genuine impact on their health. The major contributions found explaining possible further deterioration of maternal mental function are associated with episiotomies and multiparous mothers. Portugal is among the European countries that most use episiotomy in vaginal deliveries (73.0%), far beyond the recommended 10.0% ^c^. Thus, and upon increasing medical disagreement with respect to their potential effect, its use must be rethought as a causative agent of suffering and reduced quality of life in the postpartum period. Concerning multiparity, the association may report a more psychosocial explanation: multiparous women seemed to report a worse individual mental functioning, which can result from an increased complexity of roles in the family and associated impending conflicts (it not only requires the reorganization of the marital system, but also that of the previously existing parental system)^11^ ^,^ ^13^. Maternal education above 10 years of schooling was found to play a major role regarding potentially protective effects to preserve an adjusted mental functioning after delivery.

When considering the variables that seem to contribute the most for developing postpartum depression, being a migrant was significant when the cut-off is above 10. Our results also show that a previous diagnosis of depression (even if already overtaken at the time of the last pregnancy), adverse obstetric outcomes in previous pregnancies and obstetric complications during the last pregnancy (e.g., gestational hypertension) are scientifically recognized to induce accountable levels of anxiety and discomfort and to be associated with increasing odds of postpartum depression. Additionally, the association between excessive consumption of tobacco during pregnancy and the risks for maternal health is empirically understandable. This association, in this case, can also be foreseen as a hypothetic reverse causality, relying on the known association between smoking and mental illness – in which smoking acts as an escape for relief of acute and generalized anxiety. Protective associations with no medical background support were also found: having a cesarean-section (not emergent or urgent) is perceived by the mothers as a less stressful and anxiogenic event than a natural delivery – which is consistent with the cultural belief that explains such a high proportion of cesarean-section procedures in the country (36.3%), despite lacking in medical sense. Understandably, a family monthly income above the national minimum wage (485€) determines that women with more resources express a lower level of postpartum depression than those with fewer financial resources^12^.

Growing scientific knowledge is documenting the important contributions of stress in pregnancy to specific outcomes during pregnancy and birth. Stress exposures are being commonly accepted as relevant explanations to increased risk of preterm birth or having a low birth weight child^19^. Regarding our results, chronic stressors such as non-gestational conditions (e.g., anemia), obstetric complications in previous pregnancies (e.g., adverse obstetric outcomes like spontaneous miscarriage, ectopic pregnancy, stillbirth, or neonatal death) and during the last pregnancy (e.g., gestational hypertensive disorders) are key elements for perceived distress in the postpartum period. The same previous negative association was found regarding delivering by a cesarean-section and smoking less than 10 cigarettes per day during pregnancy (among smokers) as experiences that help reducing stress, confirming our explanation concerning personal perceptions and subjective meanings that influence health, irrespective of clinical sense. Maternal education seemed to play, again, a protective effect against distress, since it enables focused action and adaptation to new family roles^4^ ^,^ ^6^ ^,^ ^14^.

Lastly, when exploring social support, our findings are greatly consistent with the literature^1^ ^,^ ^2^: being a migrant is the major contribute for having a low social support. This feeling of isolation and helplessness is aggravated by a previous diagnose of depression, being multiparous and having had pregnancy and intrapartum complications in the last pregnancy (e.g., hypertensive disorders, episiotomy, and postpartum hemorrhage). Regarding maternal age, a deleterious effect may appear once migrants, who manifest an increased risk of poor social support, are themselves older (and therefore more multiparous, accumulating two potential risks). Considering variables with latent protective influences, we perceive that family income above 500€ has a major role – this aspect is consistent with the literature, since the concept of social support refers to the perceived resources available to individuals, often interceded by family financial capability^5^ ^,^ ^15^. We also found a protective effect associated with having a minor urinary infection during pregnancy that, despite no medical sense or background, may be interpreted as likely to trigger objective and emotional support and care of family and affective network.

Being a migrant appears not to be a foremost element in explaining odds for distress and depleted mental health. Despite, as these dimensions tend to be implicated in an unclear chain of mental and emotional processes regarding motherhood, potential vulnerabilities should be considered in clinical attention. This is especially true at a postpartum moment, and not only for migrants, as several gaps have been identified regarding postpartum attention to all women in public health facilities^1^ ^,^ ^2^. Clinical care is being based on “same care for all” more than equity. As socioeconomic and subjective individual experiences are achieving greater impacts on health^12^, we believe that those factors must be urgently integrated into medical care to reestablish social justice. This approach could be pertinent in helping to restore mental health in general (and maternal mental health in particular) as a priority in public health, nationally and worldwide.

## Footnotes

a Gushulak B, Pace P, Weekers J. Migration and health of migrants. In: Koller T, editor. Poverty and social exclusion in the WHO European Region: health systems respond. Copenhagen: World Health Organization; 2010. p.257-81.

b International Organization for Migration – IOM. World Migration Report 2011: communicating effectively about migration. Geneva; 2011.

c European Surveillance of Congenital Anomalies; Euro-Peristat. *The health and care of pregnant women and babies in Europe in 2010*. Paris: Euro-Peristat; 2013. (European Perinatal Health Report).
